# Matrine Alleviates Oxidative Stress and Inflammation in Colon Cancer by Activating the Nrf2 Pathway

**DOI:** 10.5152/tjg.2025.24438

**Published:** 2025-01-27

**Authors:** Yun-Fei Dong, Tao Shang

**Affiliations:** Department of Anorectal, The First Affiliated Hospital of Zhejiang Chinese Medical University (Zhejiang Provincial Hospital of Chinese Medicine), Hangzhou, Zhejiang Province, China

**Keywords:** Colon cancer, inflammation, matrine, nuclear factor erythroid 2-related factor 2 pathway, oxidative stress

## Abstract

**Background/Aims:**

Oxidative stress (OS) and inflammation are pivotal for colon cancer (CC) development, which can be regulated by the nuclear factor erythroid 2-related factor 2 (Nrf2) pathway. Matrine (MAT) can treat disorders by modulating OS and inflammation with the Nrf2 pathway. This study aimed to investigate the effects of MAT in CC.

**Materials and Methods:**

Mice pretreated with MAT were injected with 1,2-dimethylhydrazine (DMH) to induce CC. Then, colon tissue pathology was examined, and B cell lymphoma-2 (Bcl-2), Bcl-2 associated X (Bax) and antigen identified by monoclonal antibody Ki 67 (Ki-67) expressions in colon tissues were quantified. Moreover, HT-29 cells were treated with MAT and transfected with si-Nrf2 to further investigate the mechanisms of MAT on CC. Following treatment with MAT, the viability and apoptosis of HT-29 cells were detected. Furthermore, OS- and inflammation-related factors, as well as Nrf2 pathway-related expressions, were measured both in vivo and in vitro.

**Results:**

Matrine treatment alleviated DMH-induced epithelial structural changes, increased Bcl2 and Ki-67 expressions, but decreased Bax expression in the colon. In vitro, MAT inhibited viability but induced apoptosis in HT-29 cells. Both in vivo and in vitro results revealed that MAT alleviated OS and inflammation, increased Nrf2, NAD(P)H quinone dehydrogenase 1, and heme oxygenase-1 expressions, but reduced kelch-like ECH-associated protein 1 expression in CC models. However, the effects of MAT on CC were reversed by Nrf2 knockdown.

**Conclusion:**

Matrine may alleviate OS and inflammation in CC by activating the Nrf2 pathway, suggesting that MAT may be a promising strategy in CC treatment.

**Main Points**

- Matrine suppressed cancer symptoms and inflammation in colon cancer (CC) in mice.
- Matrine diminished oxidative stress (OS) in CC mice.
- Matrine activated the nuclear factor erythroid 2-related factor 2 (Nrf2) pathway in CC mice.
- Matrine inhibited viability, OS, and inflammation in CC cells by activating the Nrf2 pathway

## Introduction

Despite a decline in the overall incidence of colon cancer (CC), there has been an increase in the incidence among young people in recent years.^1^ Some researchers estimate that the incidence of CC in young patients aged 20-34 will increase by 90% by 2030.^1^ Currently, the treatment for CC includes surgery, chemotherapy, radiotherapy, and immunotherapy. However, the recurrence of CC remains a significant challenge, with a high recurrence rate and a 5-year survival rate of less than 40%.^2^

The development of CC can be attributed to a variety of factors, but a large amount of convincing evidence indicates that oxidative stress (OS) is strongly correlated with it.^3^ Oxidative stress may be involved in CC in a reactive oxygen species (ROS)-dependent manner, which may be related to the activation of phagocytes present in intestinal disease mucus to produce a large number of oxidized substances, resulting in continuous exposure of colorectal cancer to endogenous or exogenous ROS, increasing the risk of CC.^4^ Targeting antioxidant systems has been demonstrated to be a novel and effective therapeutic strategy against cancers, including CC.^5^ However, it should be noted that not only OS but also inflammation has the potential to induce tissue damage and promote the development of malignant tumors. Roessner et al^4^ have suggested that colitis may lead to epithelial cell transformation and progression to CC following inflammatory infiltration, increased ROS production, impaired antioxidant defenses, deoxyribonucleic acid (DNA) damage, and genetic and epigenetic changes. To date, anti-OS and anti-inflammatory strategies have been proven effective in alleviating cancers, and numerous drugs have been used to inhibit cancer development by alleviating OS and inflammatory conditions.

In addition to standard strategies, the utilization of traditional therapies, particularly medicinal plants, has received considerable attention in the treatment of CC. Matrine (MAT), a tetracyclic quinolizidine alkaloid isolated from the dried roots of *Sophora flavescens*, exhibits many medicinal values, such as anti-cancer, anti-inflammatory, antibacterial, and antiviral activities.^6^ Matrine is an important component of the Compound Kushen Injection, which has been used to treat various tumors in China. In vitrostudies have revealed that MAT inhibits CC development by suppressing vasculogenic mimicry, cancer cell invasion, and proliferation.^7^ In addition, Jin et al^8^ have demonstrated that MAT can induce ferroptosis in cervical cancer through activation of the piezo1 channel. However, the effects and mechanisms of MAT on CC, particularly on OS and inflammation in CC, have yet to be investigated.

Nuclear factor erythroid 2-related factor 2 (Nrf2) is a nuclear factor that is isolated and kept at low levels by an inhibitor of Nrf2 (also known as kelch-like ECH-associated protein 1 (KEAP1)) at normal ROS levels. In response to stimuli such as OS, Nrf2 will separate from KEAP1, stabilize, and translocate to the nucleus, thereby promoting the synthesis of anti-oxidant enzymes and playing a role in ROS homeostasis. Furthermore, Nrf2 is also an important regulator of inflammation and cell metabolism in cancers. Dysregulation of the Nrf2 pathway is always observed in tumor samples.^9^ Cell experiments have revealed that MAT can protect retinal ganglion cells by upregulating Nrf2 and peroxisome proliferator-activated receptor gamma coactivator 1-alpha, which, together, alleviate the OS of retinal ganglion cells.^10^ However, it is unclear whether MAT can alleviate OS and inflammation in CC by activating the Nrf2 pathway.

Therefore, in this study, mice were treated with 1,2-dimethylhydrazine (DMH) to establish CC mouse models. The CC mice were pretreated with MAT to explore the effects of MAT on CC. In addition, human CC cells HT-29 were transfected with si-Nrf2 to knockdown Nrf2 expression to further investigate whether MAT can alleviate OS and inflammation in CC by activating the Nrf2 pathway, thereby providing a novel therapeutic approach and target for CC.

## Materials and Methods

### Animals

Eighteen 6-week-old male BALB/c mice (body weight 20 ± 1.5 g) were purchased from Shanghai SLAC Laboratory Animal Co., Ltd. The mice were housed in plastic cages (3 mice/cage) for 1 week in a controlled environment with 22 ± 2°C temperature, alternating light and dark every 12 hours, and 50% humidity. All mice were given standard chow and water ad libitum. All animal experiments were conducted in accordance with the relevant regulations and approved by the Animal Experimentation Ethics Committee of Zhejiang Eyong Pharmaceutical Research and Development Center (ethics approval number: ZJEY-20231016-01; ethics approval date: October 16, 2023).

### Study Design and Colon Cancer Induction

The mice were randomly divided into 3 groups: the control group, the DMH group, and the DMH + MAT group (n = 6). Mice in the DMH + MAT group were administered MAT (50 mg/kg/d; S31035, Yuanye Biotechnology Company, China) by intraperitoneal injection for 2 weeks.^8^^,^^11^ At this time, mice in the control and DMH groups were administered an equal volume of normal saline (R22172, Yuanye Biotechnology Company, China) by intraperitoneal injection. Then, to induce CC in mice in the DMH and DMH + MAT groups, 20 mg/kg of DMH (Y56850, Yuanye Biotechnology Company, China) was dissolved in 1 mM ethylenediamine tetraacetic acid (S30020, Yuanye Biotechnology Company, China) and injected subcutaneously into the mice once a week (on the third day of the week) for 20 weeks.^12^ At this time, control mice were injected with an equal volume of ethylenediamine tetraacetic acid.

### Hematoxylin-Eosin Staining

Following the completion of DMH injection, all animals were euthanized, and the colon tissues were collected by laparotomy. For histopathological studies, the samples were fixed in formalin (R22054, Yuanye Biotechnology Company, China) for 24 hours, dehydrated in ethanol (100092683, Sinopharm Chemical Reagent Co., Ltd., China) for 1 hour, and clarified in xylene (10023418, Sinopharm Chemical Reagent Co., Ltd., China) for 1 hour, after which the tissues were embedded in paraffin and sectioned into slices. Colon sections (about 4 μm in thickness) were utilized for Hematoxylin-Eosin staining. After dewaxing and rehydrating, the samples were stained with hematoxylin and eosin (G1003, Servicebio, China). The severity of the epithelial degeneration area was evaluated under an optical microscope (E100, Nikon, Japan).

### Reactive Oxygen Species Detection

Reactive oxygen species levels in the colon samples were measured using ROS assay kits (S0033S, Beyotime, China). The collected tissue samples were chopped and suspended in phosphate-buffered saline (R22692, Yuanye Biotechnology Company, China), then incubated with 2’,7’-dichlorodihydrofluorescein diacetate (T88220, Yuanye Biotechnology Company, China) for 45 minutes. The fluorescence intensity of 2’,7’-dichlorofluorescein was then detected and exhibited as the ROS levels of the samples.

### Immunohistochemical Assay

After dewaxing and rehydration of paraffin-embedded colon sections, citric acid antigen repair buffer (G1202, Servicebio, China) was used for antigen repair. To eliminate endogenous peroxidase activity, samples were treated with 3% H_2_O_2 _(10011208, Sinopharm Chemical Reagent Co., Ltd., China). Next, the samples were covered with primary antibodies at 4°C. Following washing, horseradish peroxidase-labeled secondary antibodies (1:5000, ab97080, Abcam, UK) were used to cover the sections for another 50 minutes. After diaminobenzidine (G1211, Servicebio, China) coloration and hematoxylin staining, the slices were observed under an optical microscope. Information on the primary antibodies used in the immunohistochemical assay is displayed in Table 1.

### Cell Culture

Human CC cells HT-29 (BFN60800646) and human normal colon epithelial cells NCM460 (BFN608006385) were obtained from the American Type Culture Collection (USA). HT-29 cells and NCM460 cells were cultured in Dulbecco’s modified Eagle medium (SH30023.01, HyClone, USA) or RPMI-1640 medium (SH30027.02, Hyclone, USA), respectively. All the media were supplemented with 10% fetal bovine serum (SH30087.01, HyClone, USA), 100 U/mL penicillin (SH30010, HyClone, USA), and 100 µg/mL streptomycin (S9137, Sigma, USA).

### Cell Counting Kit-8 Assay

HT-29 cells and NCM460 cells were seeded in 96-well plates (3599, Corning, USA). After treatment with MAT with the indicated concentrations (0-6.4 mM) and time points (24-72 hours), CCK-8 solutions (C0039, Beyotime, China) were added to the wells.^13^ After incubation for 2 hours, the absorbance of the wells was read and recorded using a microplate reader (CMaxPlus, MD, USA) at 450 nm. Finally, cell viability was calculated, and the most appropriate treatment concentration and time of MAT for HT-29 cells were determined for the following experiments.

### Cell Transfection and Treatment

Small interfering ribonucleic acid (RNA) against Nrf2 (si-Nrf2) and negative control (si-NC) were obtained from GenePharma Co. Ltd. (China). HT-29 cells were cultured in 6-well plates (353046, Corning, USA) and then transfected with si-Nrf2 or si-NC using Lipofectamine 2000 reagent (11668030, Invitrogen, USA). After 48 hours of transfection, the transfection efficiency was verified by quantitative polymerase chain reaction (qPCR). Then, HT-29 cells were divided into 4 groups, including a control group (untransfected HT-29 cells that received no treatment), MAT group (untransfected HT-29 cells treated with 3.2 mM MAT for 48 hours), MAT+si-NC group (HT-29 cells transfected with si-NC treated with 3.2 mM MAT for 48 hours), and MAT + si-Nrf2 group (HT-29 cells transfected with si-Nrf2 treated with 3.2 mM MAT for 48 hours).

### Quantitative Polymerase Chain Reaction

Total RNA of colon tissues or HT-29 cells was extracted using RNA extraction kits (AG21024, Accurate Biology). Subsequently, the total RNA was reversed transcribed into complementary DNA by reverse transcription kits (CW2569M, CWBIO, China). SYBR Green qPCR kits (11201ES03, Yeasen, China) were used to perform qPCR, and the primer sequences employed in qPCR are presented in Table 2. The expressions of the target genes were quantified by the 2^−ΔΔCT^ method.

### Methylthiazolyldiphenyl-Tetrazolium Bromide Assay

After receiving MAT treatment, HT-29 cells were harvested and plated in 96-well plates. Then, MTT solution (5 mg/mL, ST316, Beyotime, China) was added to incubate the cells in the dark for 4 hours. Thereafter, formazan was solubilized by dimethyl sulfoxide (D8418, Sigma, USA), and the absorbance was detected at 490 nm with a microplate reader.

### Flow Cytometry Analysis

Apoptosis detection kits (556547, BD, USA) were used to detect apoptotic changes in HT-29 cells. After treatment with MAT, HT-29 cells were harvested, rinsed, and centrifuged. The cell pellets were then suspended in binding buffers and incubated with Annexin V-fluorescein isothiocyanate and propidium iodide solution. Finally, the apoptosis rates of HT-29 cells were quantified using a flow cytometer (NovoCyte, Agilent, USA).

### Assessment of Oxidative Stress Status

Colon tissues were homogenized in phosphate buffer saline, and HT-29 cells were lysed by cell lysis solutions. After obtaining tissue homogenates and cell lysates, the activities of superoxide dismutase (SOD, tissue homogenates: A001-3-2; cell lysates: A001-1) and malondialdehyde (MDA, tissue homogenates: A003-1; cell lysates: A003-4-1) in tissue homogenates and cell lysates, as well as the activities of catalase (CAT, A007-1-1), glutathione peroxidase (GPx, A005-1-2), glutathione reductase (GSR, A062-1-1), glutathione S-transferase (GST, BC0350) in tissue homogenates, were determined using commercial kits. The GST kits were purchased from Beijing Solaibao Technology Co., Ltd. (China), and other kits were supplied by Jiancheng Bioengineering Institute (China).

### Enzyme-Linked Immunosorbent Assay

After treatments, colon samples were homogenized and centrifuged to collect the supernatant. HT-29 cells were also centrifuged to collect the supernatant. The levels of interleukin-6 (IL-6, tissue homogenates: MM-0163M2; cell lysates: RX106126H), tumor necrosis factor-α (TNF-α, tissue homogenates: MM-0132M2; cell lysates: RX104793H) in colon tissues and HT-29 cells, as well as the levels of inducible nitric oxide synthase (iNOS, MM-0454M2), prostaglandin E2 (PGE-2, E-EL-0034c) and cyclooxygenase-2 (COX-2, MM-0356M2) in colon tissues were quantified using commercial ELISA kits. Except for the PGE-2 ELISA kits from Elabscience Biotechnology Co., Ltd. (China), human IL-6 and TNF-α ELISA kits were provided by Ruixin Biotech (China), and others were purchased from Meimian Bioscience Co., Ltd. (China).

### Western Blot Assay

The total protein of colon samples and HT-29 cells was isolated by radioimmunoprecipitation assay buffer (P0013B, Beyotime, China) and quantified by bicinchoninic acid assay kits (P0012, Beyotime, China). Then, the protein was separated by sodium dodecyl sulfate-polyacrylamide gel electrophoresis (P0012A, Beyotime, China) and transferred to polyvinylidene fluoride membranes (10600023, GE Healthcare Life, USA). After treatment with 5% skimmed milk (P0216, Beyotime, China), the membranes were incubated with primary antibodies and secondary antibodies (1:6000, 7074, CST, USA) in turn. Finally, the membranes were visualized using enhanced chemiluminescence reagents (610020-9Q, Clinx, China). The band densities were analyzed by ImageJ software (ImageJ 1.52, National Institutes of Health, USA). The information on the primary antibodies used in the Western blot assays is exhibited in Table 3.

### Statistical Analysis

SPSS 20.0 statistical software (IBM SPSS Corp.; Armonk, NY, USA) was used to analyze the data. All data were presented as mean ± standard deviation. One-way analysis of variance with Tukey’s test was used for measurement data among multiple groups. *P *< .05 was considered statistically significant.

## Results

### Matrine Suppressed Cancer Symptoms and Inflammation in 1,2-Dimethylhydrazine-Administered Mice

**Figure 1.**
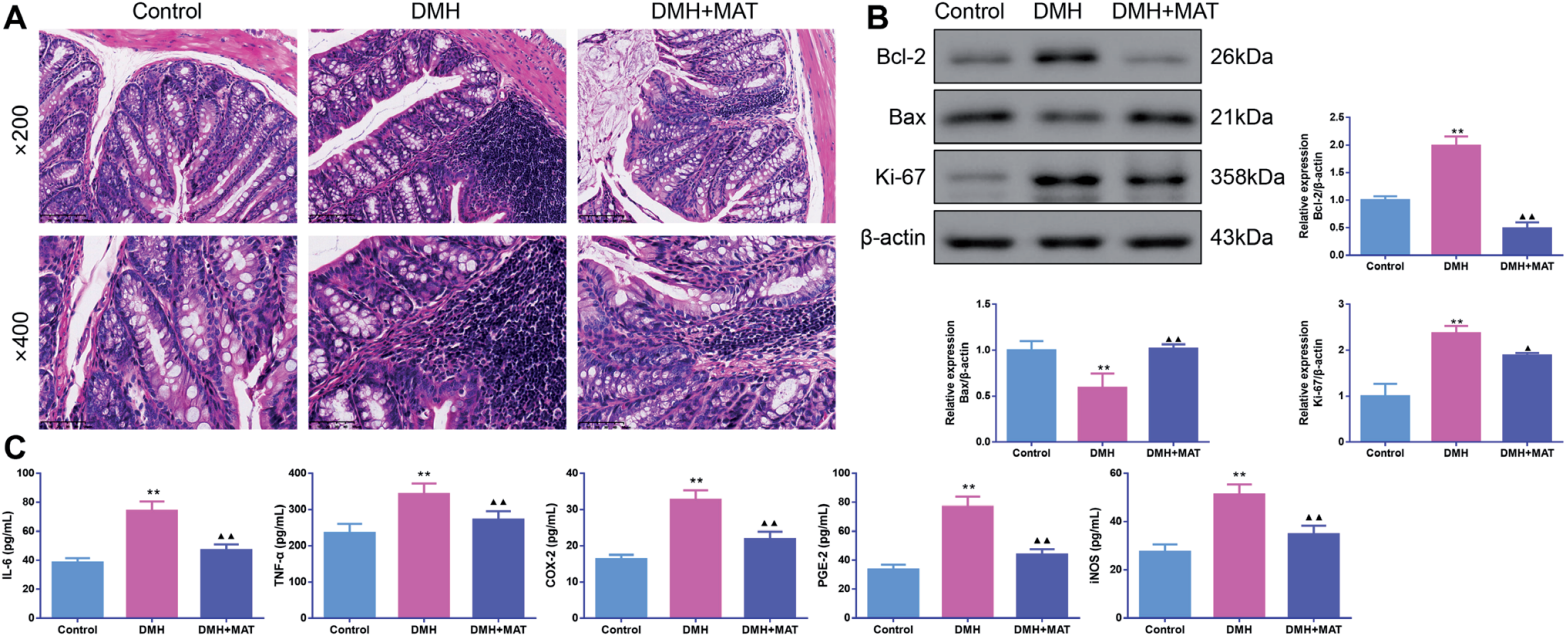
Matrine alleviated cancer symptoms and inflammation in 1,2-dimethylhydrazine (DMH)-administered mice. (A) Colon tissue pathology was assessed by hematoxylin-eosin staining. Scale bar: 100 μm (magnification: ×200); scale bar: 50 μm (magnification: ×400); (B) Western blot assays were used to detect B cell lymphoma-2 (Bcl-2), Bcl-2 associated X (Bax) and antigen identified by monoclonal antibody Ki 67 (Ki-67) protein expression in colon tissues. n = 3. (C) Inflammatory factor levels of interleukin-6 (IL-6), tumor necrosis factor-α (TNF-α), cyclooxygenase-2 (COX-2), prostaglandin E2 (PGE-2), and inducible nitric oxide synthase (iNOS) in colon tissue of mice were measured using enzyme-linked immunosorbent assay (ELISA). n = 6. Results were presented as mean ± standard deviation (SD). *P < .05 and **P < .01 vs. Control group; ^▲^*P* < .05 and ^▲▲^*P* < .01 vs. DMH group.

As depicted in Figure 1A, colon tissue stained with HE revealed that the colon tissue structure of control mice had clear layers without obvious abnormalities. In contrast, obvious inflammatory cell infiltration, epithelium destruction and crypts were found in DMH mice. Compared with DMH mice, the epithelial lesion and the degeneration degree were reduced in the DMH + MAT group. Moreover, Bcl-2 and Ki-67 protein levels were increased, but the Bcl-2 associated X (Bax) protein level was decreased in DMH-treated mice (*P *< .01). However, MAT administration effectively reversed these conditions (*P *< .05, Figure 1B). Furthermore, increased levels of IL-6, TNF-α, COX-2, PGE-2, and iNOS were observed in the colon tissues of the DMH group (*P *< .01). In contrast to the DMH group, the MAT group showed a significant decrease in the levels of the above pro-inflammatory mediators (*P *< .01, Figure 1C).

### Matrine Diminished Oxidative Stress in 1,2-Dimethylhydrazine-Administered Mice

**Figure 2.**
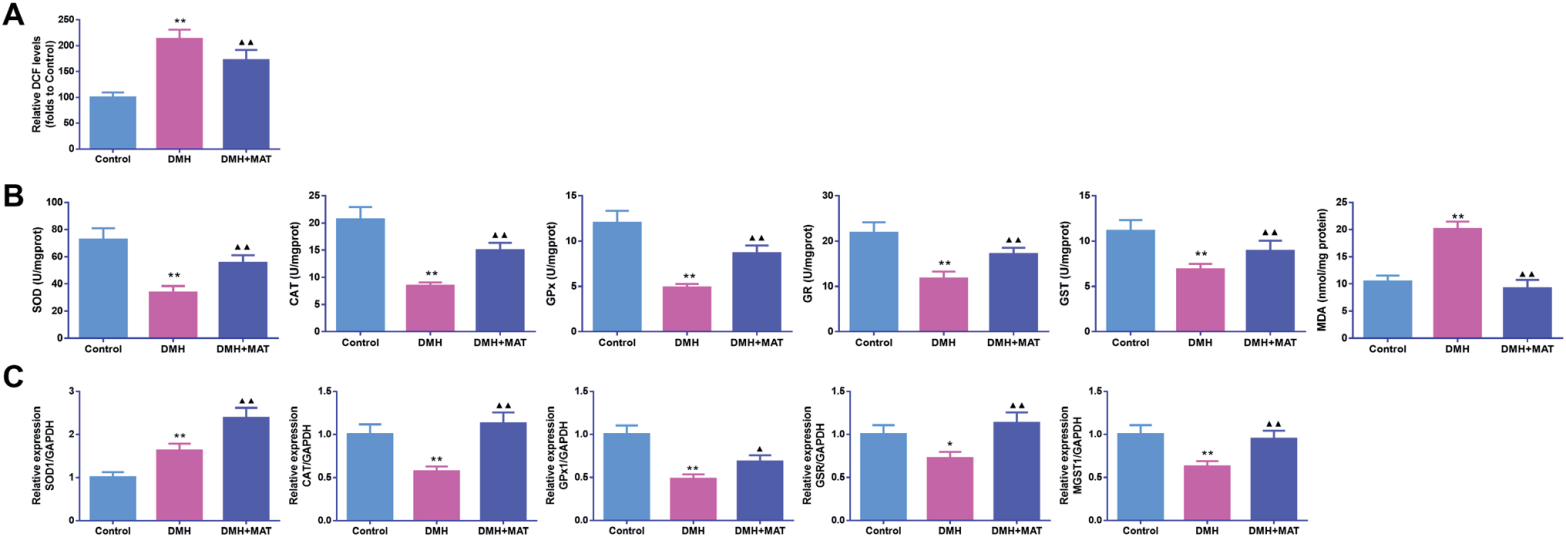
Matrine inhibited oxidative stress for DMH-administered mice. (A) The reactive oxygen species (ROS) levels in colon tissues were measured by 2,7-dichlorofulson diacetate assay. n = 6. (B) The activities of superoxide dismutase (SOD), catalase (CAT), glutathione peroxidase (GPx), glutathione reductase (GSR), glutathione S-transferase (GST), and malondialdehyde (MDA) in colon tissues were determined by commercial kits. n = 6. (C) The messenger ribonucleic acid levels of SOD (SOD1), CAT, GPx (GPx1), GSR, and GST (microsomal glutathione S-transferase 1 (MGST1)) were measured using quantitative polymerase chain reaction (qPCR). n = 3. Results were presented as mean ± SD. *P < .05 and **P < .01 vs. Control group; ^▲^*P* < .05 and ^▲▲^*P* < .01 vs. DMH group.

As presented in Figure 2A, DMH induced ROS generation in the colon tissues of the DMH group (*P *< .01). Interestingly, after MAT treatment, the ROS level in the colon tissues of the DMH + MAT group declined (*P *< .01). Furthermore, as shown in Figure 2B, relative to the control mice, the activities of SOD, CAT, GPx, GSR, and GST were diminished, whereas the activity of MDA was augmented in the DMH group (*P *< .01). Nevertheless, MAT administration reversed the activities of the above OS-related indicators (*P *< .01). The messenger RNA (mRNA) levels of OS-related indicators were further measured by qPCR. As shown in Figure 2C, except SOD (SOD1) mRNA, the expressions of CAT, GPx (GPx1), GSR and GST (microsomal glutathione S-transferase 1 (MGST1)) mRNA were consistent with the activity results. Superoxide dismutase mRNA level was increased after DMH induction, while after MAT treatment, SOD mRNA level exhibited a further increase in the DMH + MAT group (*P *< .01).

### Matrine Activated the Nuclear Factor Erythroid 2-Related Factor 2 Pathway in 1,2-Dimethylhydrazine-Administered Mice

**Figure 3.**
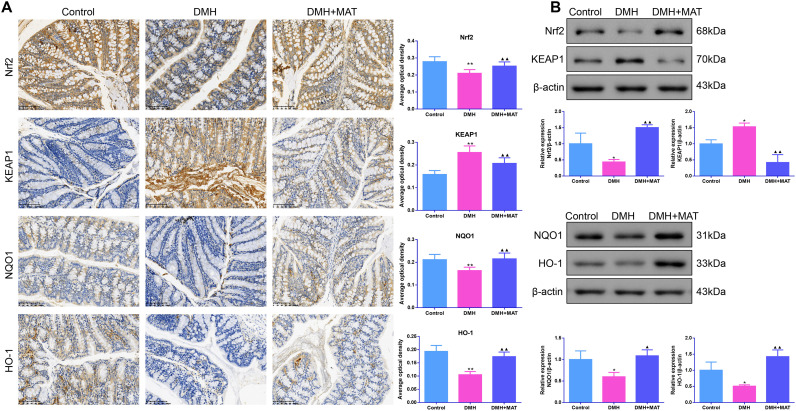
MAT activated the nuclear factor erythroid 2-related factor 2 (Nrf2) pathway for DMH-administered mice. (A) Immunohistochemical assay was used to determine the expression levels of Nrf2, kelch like ECH associated protein 1 (KEAP1), NAD(P)H quinone dehydrogenase 1 (NQO1) and heme oxygenase-1 (HO-1) proteins in colon tissues. Scale bar: 100 μm (magnification: ×200), n = 6. (B) Western blot assays were used to determine the expression levels of Nrf2, KEAP1, NQO1, and HO-1 proteins in colon tissues. n = 3. Results were presented as mean ± SD. *P < .05 and **P < .01 vs. Control group; ^▲^*P* < .05 and ^▲▲^*P* < .01 vs. DMH group.

Immunohistochemistry results are presented in Figure 3A. Relative to the control group, Nrf2, NAD(P)H quinone dehydrogenase 1 (NQO1) and heme oxygenase-1 (HO-1) expressions were decreased, whereas KEAP1 expression was increased in the colon of the DMH group (*P *< .01). However, after administration with MAT, Nrf2, NQO1, and HO-1 expressions were upregulated, whereas KEAP1 expression was downregulated in the colon (*P *< .01). Subsequently, Nrf2 pathway-associated protein levels were further examined using Western blot assays, and the results were consistent with those observed in the immunohistochemical assay (Figure 3B).

### Matrine Inhibited Viability, Oxidative Stress, and Inflammation of Colon Cancer Cells by Activating the Nuclear Factor Erythroid 2-Related Factor 2 Pathway

**Figure 4.**
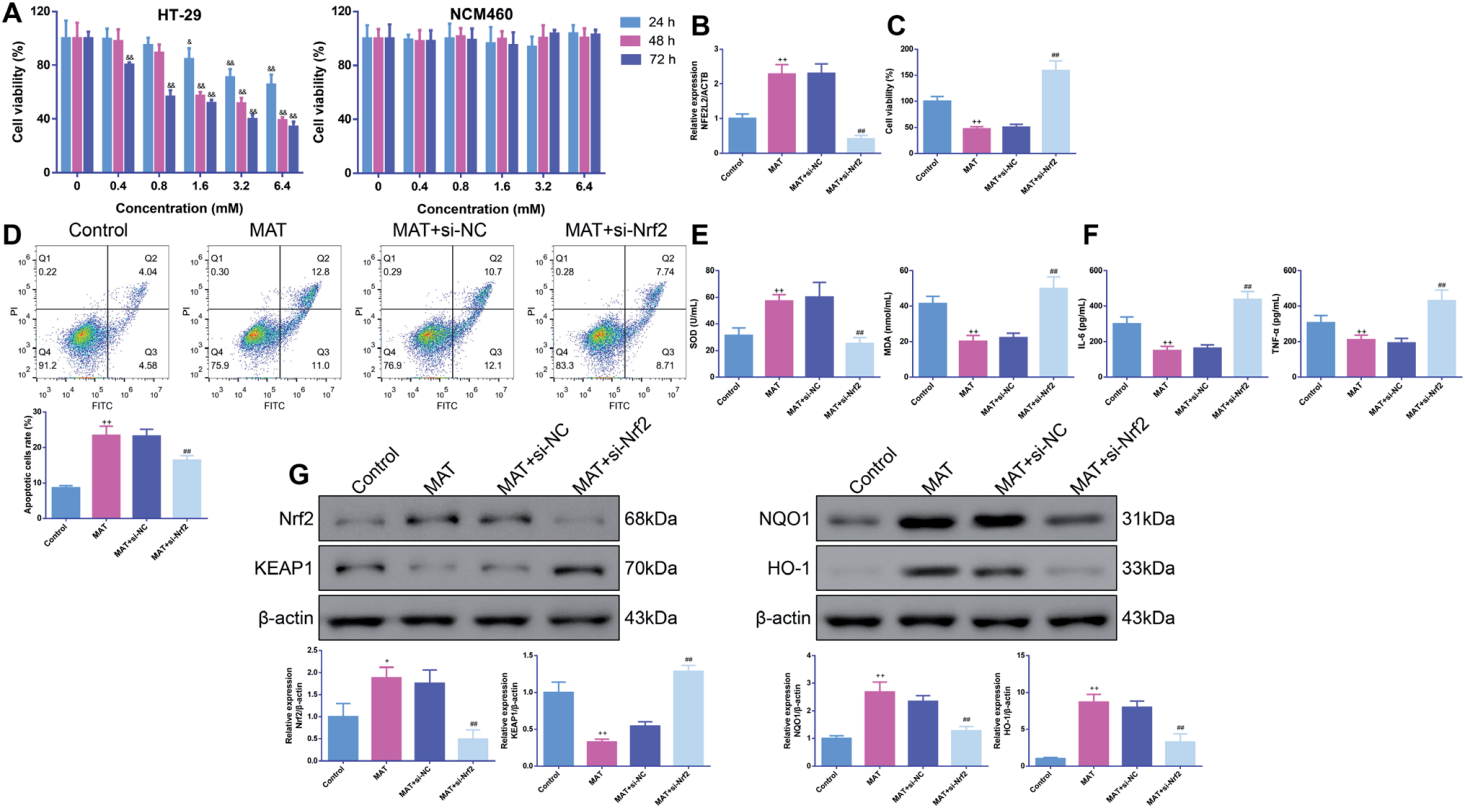
MAT inhibited viability, oxidative stress, and inflammation of colon cancer cells by activating the Nrf2 pathway. (A) Cell counting kit-8 assay was used to detect the cell viability of NCM460 cells and HT-29 cells. n = 6. (B) The transfection efficiency of si-Nrf2 was confirmed by qPCR. n = 3. (C) Methylthiazolyldiphenyl-tetrazolium bromide assay was used to measure HT-29 cell viability. n = 6. (D) Flow cytometry analysis was employed to test the apoptosis of HT-29 cells. n = 3. (E) The activities of SOD and MDA in HT-29 cells were quantified. n = 6. (F) The levels of IL-6 and TNF-α were tested by ELISA. n = 6. (G) Western blot assays were used to determine the expression levels of Nrf2, KEAP1, NQO1, and HO-1 proteins in HT-29 cells. n = 3. Results were presented as mean ± SD. ^+^*P* < .05 and ^++^*P* < .01 vs. Control group; ^#^*P* < .05 and ^##^*P* < .01 vs. MAT + si-NC group.

The CCK-8 results demonstrated that the treatment of human CC cells HT-29 with MAT at the concentrations of 1.6 mM, 3.2 mM, and 6.4 mM could inhibit cell viability, irrespective of the treatment time (24 hours, 48 hours or 72 hours) (*P *< .05). Therefore, the subsequent experiment was conducted using a MAT concentration of 3.2 mM and a treatment time of 48 hours, which showed no toxicity to human normal colon epithelial cells NCM460 (Figure 4A). To further confirm the mechanisms of MAT on CC, HT-29 cells were transfected with si-Nrf2. As exhibited in Figure 4B, MAT was observed to increase Nrf2 (NFE2L2) mRNA levels in HT-29 cells; this effect was counteracted by Nrf2 knockdown (*P *< .05). Furthermore, treating HT-29 cells with MAT reduced cell viability and promoted apoptosis, but these changes were counteracted by transfection with si-Nrf2 (*P *< .05, Figure 4C and D). Furthermore, compared with the control cells, MAT was observed to increase SOD activity but decreased MDA activity as well as IL-6 and TNF-α levels for HT-29 cells (*P *< .05). Nevertheless, these effects were reversed by Nrf2 knockdown (*P *< .05, Figure 4E and F).

Next, Western blot assays were performed to quantify Nrf2 pathway-related protein levels. As exhibited in Figure 4G, relative to the control cells, MAT was shown to upregulate Nrf2, NQO1, and HO-1 protein expression but downregulated KEAP1 protein expression in HT-29 cells, which was consistent with those observed in the in vivoexperiments (*P *< .05). However, after Nrf2 knockdown, Nrf2, NQO1, and HO-1 protein expression were decreased, while KEAP1 protein expression was increased in HT-29 cells (*P *< .01).

## Discussion

Despite a continued decline in mortality rates, CC remains the leading cause of morbidity and mortality among digestive system cancers, and the high recurrence rate of CC after treatment is still worrisome. Recently, the functions of medicinal plants as well as their main compounds on cancer have been widely studied, in which MAT has been proven to suppress the growth and induce apoptosis in various cancer cells.^14^ The inhibitory effects of MAT on CC cells have been demonstrated in several published studies.^15^ Given that DMH-induced experimental CC has similar histological morphology and anatomical structure to human CC, DMH is always used to induce CC in mice to investigate the functions of novel drugs for CC.^12^ In this study, we discovered that MAT could alleviate OS and inflammation in CC by activating the Nrf2 pathway.

Matrine, a principal active compound in the traditional Chinese herb *S. flavescens*, has been authorized by the China Food and Drug Administration for the prevention and treatment of cancer cachexia. Matrine has shown inhibitory effects on various types of tumors. Consistently, in this study, we observed that MAT not only alleviated DMH-induced epithelial structural changes but also decreased Bcl-2 and Ki-67 and increased Bax expressions in CC. In addition, MAT decreased viability but induced apoptosis in HT-29 cells. Taken together, these data suggested that MAT had the potential to alleviate CC.

It is widely accepted that inflammation is pivotal in the development of CC, and that inflammation can initiate and promote colon carcinogenesis by inducing DNA damage or epigenetic changes. Zhao et al^16^ have demonstrated that the IL-6 cytokine family signal transducer (IL-6ST) is significantly elevated in CC tissues, and the specificity and sensitivity of IL-6ST for predicting CC are high; more importantly, they have found that IL-6ST knockdown decreases CC cell viability, promotes the ferroptosis phenotype, and increases iron accumulation and ROS production. In addition, given the extensive infiltration of diverse immune cells within the tumor microenvironment, it is widely accepted that inflammation exerts pivotal functions in many aspects of cancers. Scholars have reported that inflammation is a risk factor for CC.^17^ Hence, inhibiting the levels of inflammatory biomolecules may influence the occurrence and development of CC.

Matrine has anti-inflammatory properties in many diseases. To further evaluate the anti-inflammatory functions of MAT on CC, ELISA was applied in the study to measure inflammatory biomolecule levels. Interleukin-6 trans-signaling in epithelial cells has been identified as a critical promoter for the development and growth of associated cancers, while TNF-α has been proven to contribute to the development of associated cancers by regulating the infiltration of inflammatory cells, neutrophils, and macrophages.^18^ Interestingly, IL-6 and TNF-α have interactions and coordination in the development of CC.^19^ In our study, IL-6 and TNF-α levels were upregulated in DMH-induced CC tissues, while MAT treatment was observed to inhibit the IL-6 and TNF-α levels, indicating that MAT played a counter role in the inflammatory process of DMH-induced CC. In addition, COX-2 and PGE-2 are involved in vasodilation, thereby leading to inflammation, while COX-2-derived PGE-2 stimulates macrophages to produce pro-inflammatory chemokines, thereby contributing to associated carcinogenesis.^20^^,^^21^ Our study found that MAT inhibited the elevations of COX-2 and PGE-2 in DMH-induced CC, suggesting multiple inhibitory effects of MAT on inflammation in CC. It is worth noting that iNOS is ubiquitously overexpressed in inflammation-associated CC, and the development of iNOS inhibitors has been considered to contribute to targeted therapies for CC.^22^ The significant inhibitory effects of MAT on iNOS were also observed in our study.

Apart from inflammation, OS is also an important factor in the development and progression of CC. Matrine has been demonstrated to ameliorate colitis by inhibiting OS. A published study has reported that heightened OS is observed in the colon of DMH-induced mice.^23^ Furthermore, OS has also been reported to be a key factor in excessive cell apoptosis. Yang et al^24^ have demonstrated the close relationship between Sirtuin 1, OS-related genes, apoptosis-related genes, and inflammation-related genes through protein−protein interaction networks. Reactive oxygen species are a crucial indicator for OS. Reactive oxygen species levels in colon tissues were measured in our study. An increase in ROS levels was observed in DMH-induced CC tissues, while MAT treatment reduced the increased ROS levels in DMH-induced CC tissues, indicating that MAT may be an effective ROS inhibitor. Endogenous antioxidants, particularly antioxidases as the first line of antioxidant defenses, counteract OS to maintain a redox balance, thereby reducing the risk of diseases and cancers. Decreased activities of antioxidant enzymes have been verified in CC patients.^25^ Consistently, decreased activities of SOD, CAT, GPx, GSR, and GST were also observed in CC tissues of DMH mice in our study, while MAT treatment resulted in an increase in antioxidant activities compared to the DMH group. However, special data were observed in the antioxidant experiments: compared to the control group, SOD activity decreased but SOD mRNA levels increased in the DMH group. In fact, Zińczuk et al^26^ have demonstrated that the SOD level is obviously higher in tumor tissues than in normal colonic mucosa tissues, while CAT activity depended on the intensity of inflammatory infiltration. Therefore, how the levels of antioxidant enzymes change in CC needs to be further determined, but it is confirmed that antioxidase levels can be used to evaluate tumor progression, and MAT could promote the expressions and activities of antioxidases.

Nuclear factor erythroid 2-related factor 2 is often described as a “necessary evil” as it has been shown to promote antioxidant responses and chemo-resistance.^27^^,28^ Moreover, activation of the Nrf2 pathway regulates the expression levels of hundreds of gene products that affect inflammation and OS, while the risk of CC increases when Nrf2 is inhibited. Our study revealed that in DMH-induced CC, Nrf2 expression was suppressed but KEAP-1 levels were increased. KEAP-1, a cysteine-rich protein, binds to Nrf2 and anchors it to the cytoskeleton, which inhibits Nrf2 translocation to the nucleus and prevents it from entering the promoter. Therefore, it is not difficult to explain that the decreased Nrf2 expression level observed in the DMH group was due to the increased KEAP-1 expression level in CC. When Nrf2 is translocated to the nucleus, it binds to antioxidant response elements in the promoters of key antioxidant genes (e.g., HO-1, NQO1) and activates their transcription.^29^^,^^30^ In addition, the study conducted by Han et al^30^ has shown that the change in expression of Nrf2 is consistent with that of HO-1 and NQO1 in rats, both in K_2_Cr_2_O_7_-induced lung injury and subsequently treated with melatonin. Our experiments found that HO-1 and NQO1 expression levels were upregulated after MAT treatment, indicating that MAT activated the Nrf2 pathway. Furthermore, it should be noted that the inhibitory effects of MAT on inflammation and OS for CC were reversed by Nrf2 knockdownin vitro, indicating that MAT may alleviate inflammation and OS in CC by activating the Nrf2 pathway.

In summary, our study data provided evidence that MAT could alleviate OS and inflammation in CC both in vivo andin vitro by activating the Nrf2 pathway. Therefore, MAT has the potential to treat CC, and the Nrf2 pathway could be a potential therapeutic target for CC.

## Figures and Tables

**Table 1.** Primary Antibody Information for the Immunohistochemical Assay

Antibody	Source	Cat. No.	Dilutions
Nrf2	Affinity	AF0639	1:200
KEAP1	Affinity	DF6637	1:200
NQO1	Affinity	DF6437	1:200
HO-1	Affinity	AF5393	1:200

**Table 2.** Quantitative Polymerase Chain Reaction Primers

Gene	Forward Primer	Reverse Primer	GenBank Accession	Product Size (bp)
Mouse SOD1	AACCAGTTGTGTTGTCAGGAC	CCACCATGTTTCTTAGAGTGAGG	NM_011434	139
Mouse CAT	GTCCGATTCTCCACAGTCGC	CGCTGAACAAGAAAGTAACCTG	NM_009804	274
Mouse GPx1	CACAGTCCACCGTGTATGCC	AAGTTGGGCTCGAACCCACC	NM_008160	292
Mouse GSR	GACACCTCTTCCTTCGACTACC	CCCAGCTTGTGACTCTCCAC	NM_010344	116
Mouse MGST1	CTCAGGCAGCTCATGGACAAT	GTTATCCTCTGGAATGCGGTC	NM_019946	110
Mouse GAPDH	AGGTCGGTGTGAACGGATTTG	GGGGTCGTTGATGGCAACA	NM_008084	95
Human NFE2L2	CAGTCAGCGACGGAAAGAGT	TCTTGTTTGCTGCAGGGAGT	NM_001145412	1645
Human ACTB	CATGTACGTTGCTATCCAGGC	CTCCTTAATGTCACGCACGAT	NM_001101	250

**Table 3.** Primary Antibody Information for Western Blot Assay

Antibody	Source	Cat. No.	Dilutions
Nrf2	Affinity	AF0639	1:1000
KEAP1	Affinity	AF5266	1:1000
NQO1	Affinity	DF6437	1:1000
HO-1	Affinity	AF5393	1:1000
Bcl-2	Affinity	AF6139	1:1000
Bax	Affinity	AF0120	1:1000
Ki-67	Affinity	AF0198	1:1000

## Data Availability

All data included in this study are available upon request by contact with the corresponding author.
